# A new era of cancer immunotherapy: combining revolutionary technologies for enhanced CAR-M therapy

**DOI:** 10.1186/s12943-024-02032-9

**Published:** 2024-06-01

**Authors:** Na Li, Shinan Geng, Zhen-zhen Dong, Ying Jin, Hangjie Ying, Hung-Wing Li, Liyun Shi

**Affiliations:** 1 Key lab of Artificial Organs and Computational Medicine, Institute of Translational Medicine, Zhejiang Shuren University, Hangzhou, Zhejiang 310015 China; 2 Department of Immunology, Nanjing University of Chinese Medicine, Nanjing, Jiangsu 210023 China; 3 Department of Chemistry, The Chinese University of Hong Kong, Shatin, Hong Kong China; 4 Hangzhou Institute of Medicine (HIM), Zhejiang Caner Hospital, Chinese Academy of Sciences, Hangzhou, Zhejiang 310022 China

**Keywords:** Macrophage, Chimeric antigen receptor, CAR-M therapy, Biomaterial gene delivery

## Abstract

Significant advancements have been made in the application of chimeric antigen receptor (CAR)-T treatment for blood cancers during the previous ten years. However, its effectiveness in treating solid tumors is still lacking, necessitating the exploration of alternative immunotherapies that can overcome the significant challenges faced by current CAR-T cells. CAR-based immunotherapy against solid tumors shows promise with the emergence of macrophages, which possess robust phagocytic abilities, antigen-presenting functions, and the ability to modify the tumor microenvironment and stimulate adaptive responses. This paper presents a thorough examination of the latest progress in CAR-M therapy, covering both basic scientific studies and clinical trials. This study examines the primary obstacles hindering the realization of the complete potential of CAR-M therapy, as well as the potential strategies that can be employed to overcome these hurdles. With the emergence of revolutionary technologies like in situ genetic modification, synthetic biology techniques, and biomaterial-supported gene transfer, which provide a wider array of resources for manipulating tumor-associated macrophages, we suggest that combining these advanced methods will result in the creation of a new era of CAR-M therapy that demonstrates improved efficacy, safety, and availability.

**Graphical Abstract:**

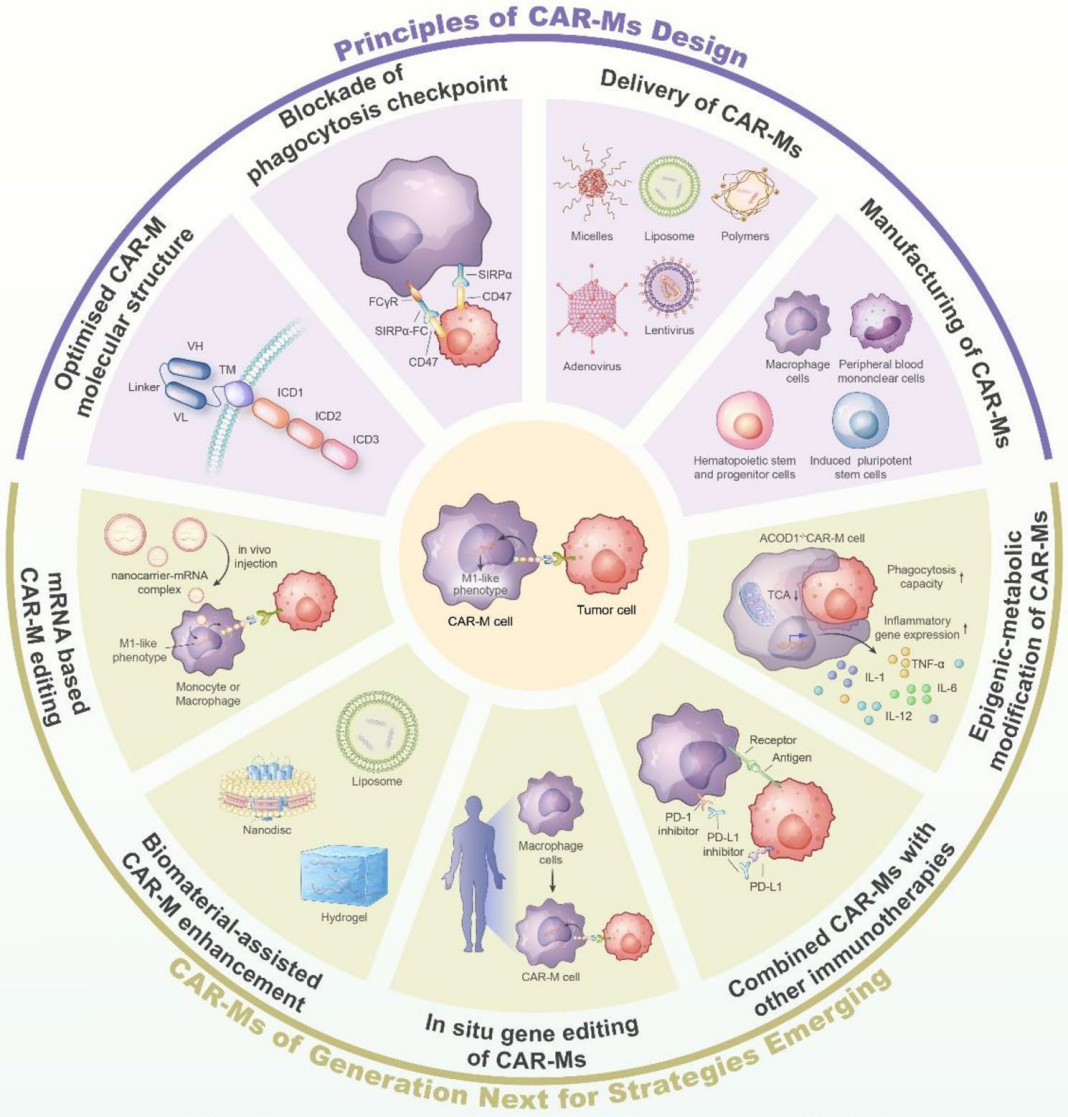

## Background

CARs, which are artificial transmembrane receptors, are implanted into immune cells to eliminate cancer cells. CAR-T cell therapy, which utilizes advancements in tumor immunobiology and cell engineering technologies, has been successfully implemented in clinical practice [1, 2]. In the past ten years, there has been a remarkable achievement in the use of CAR-T cells for the treatment of blood cancers, particularly acute lymphoblastic leukemia (ALL), lymphomas, and plasma cell myeloma (PCM) [3, 4]. However, some critical issues such as relatively low efficacy, short-term persistence and potentially the off-target effect substantially impede broader application of CAR-T cells [5–7]. Moreover, the characteristics linked to solid neoplasms, like the compact tissue structure, the diverse tumor cells, and the immune system-suppressing microenvironment, greatly restrict the clinical effectiveness of CAR-T cells [8–10]. Efforts are being made to further optimize CAR-T designing strategy, and alternatively, immune cells beyond T cells are being explored as a promising CAR-cell regime to overcome conventional adoption limitations [11].

Macrophages are known as major innate immune cells that exert multiple functions in immunity, inflammation, repairing and regeneration, etc. They represent the largest population of immune infiltrates in tumors, with nearly 50% of cell mass in most solid cancers [12]. Tumor-associated macrophages (TAMs) are highly plastic and varied, playing a pivotal role in nearly all stages of tumor development and progression. Notably, macrophages possess a plethora of properties involving potent phagocytic ability, antigen-presenting activity, secreting cytokines and chemokines, and penetrating dense tissue and accumulating in tumors [13, 14]. These attributes make macrophages a potential candidate that can be manipulated for CAR immunotherapy [15].

As early as 2006, Biglari et al. did engineer human monocytes with CEA-targeting CAR molecules and demonstrated the feasibility and safety of this therapy [16]. Since then, endeavors have been underway to develop and optimize CAR-engineered macrophages. The initial findings have shown the potential of CAR-M treatments in the management of both blood and non-blood tumors, culminating in the approval of two CAR-Ms (CT-0508 and MCY-M11) by the FDA for participation in clinical trials [17, 18]. Despite the progress achieved, current CAR-M therapy is just in its infancy and some major concerns such as limited cell resources, the resistance of gene transfer, and potentially inflammatory pathology greatly obstruct its application as a potent cancer immunetherapy [12]. With the integration of human iPSCs preparation, genetic editing technology, and biomaterial delivery, new-generation CAR-Ms equipped with specific tumor antigen recognition units, feasible genetic modification, improved expansion capability, and controllable safety are eagerly expected [19, 20].

This review provides an in-depth analysis of the present state of CAR-M research, with a specific emphasis on CAR development and the engineering of macrophages. It discusses the benefits and drawbacks of CAR-M therapy, as well as the most recent progress in fundamental and clinical investigations related to CAR-M therapy. We also discuss the emerging new technologies involving biomaterial-assisted CAR delivery, in situ gene editing, and combined therapies that are instructive to support the next generation of CAR-Ms, a more efficient, feasible, and accessible immunotherapy for malignancy.

## The complicated roles of TAMs

For a long time, macrophages have been acknowledged as powerful cells that can engulf and destroy invading pathogens or cellular debris. These cells possess various surface receptors, including mannose receptor (MR), scavenger receptor (SR), Toll-like receptor (TLR), and immunoglobulin receptor, which enable them to identify and respond to the presence of harmful microorganisms or damaged cells. Macrophages eliminate and remove internalized pathogens and outdated cellular components through the secretion of reactive oxygen intermediates, reactive nitrogen intermediates, and lysozyme [21]. In addition to its canonical phagocytic capability, macrophages fulfill a wide range of activities that have either supportive or inhibitory effects on cancer development and progression. Increasing proof indicates that within a designated ‘hot’ tumor microenvironment (TME), macrophages tend to discharge the pro-inflammatory and chemokines after engulfing cancer cells, which then allure and exhibit the antigenic signals to activate CD4^+^ T cells and cytotoxic CD8^+^ T cells. These proinflammatory macrophages are traditionally catabolized as M1 type that are generally defined as anti-tumor subsets.

Nevertheless, tumor-associated macrophages (TAMs) are known highly pliable and easily educated by tumor-derived factors, which frequently promotes the phenotypic shift of intratumoral macrophages from the tumor-favoring type to the tumor-supportive one, namely M2 [22, 23]. M2 macrophages are a type of reparative macrophage subset known for their production of anti-inflammatory cytokines like IL-10, IL-4, and transforming growth factor-β (TGF-β). They also secrete vascular endothelial growth factors (VEGF) and matrix metalloproteinases (MMPs) to facilitate tumor invasion and metastasis [24, 25]. It is currently known that M1-like TAMs are commonly dominant in early tumor stages, and M2-like TAMs become prominent in aggressive cancers. While the M1/M2 distinction aids in comprehending the functional significance of macrophages in cancer development, it is important to acknowledge the considerable heterogeneity within the intratumoral macrophage population. This heterogeneity encompasses the presence of M1, M2, and/or macrophages with mixed phenotypes coexisting within tumor niches [26, 27].

Given their functional significance, multiple regulatory roles, and predominance in tumors, the macrophage-targeting strategies, to deplete, reverse, or remold TAMs, are now intensely explored. Inspired by the success of CAR-T therapeutics in treating patients with blood malignancy, the researchers recently introduced the CAR technology into macrophages, endowing them with specific phagocytosis, enhanced antigen-presenting capability, and TME-regulatory properties. CAR-M therapy has emerged as a competitive candidate for future immunotherapy of solid tumors [28, 29].

## The progress of CAR-M research

**Fig. 1:**
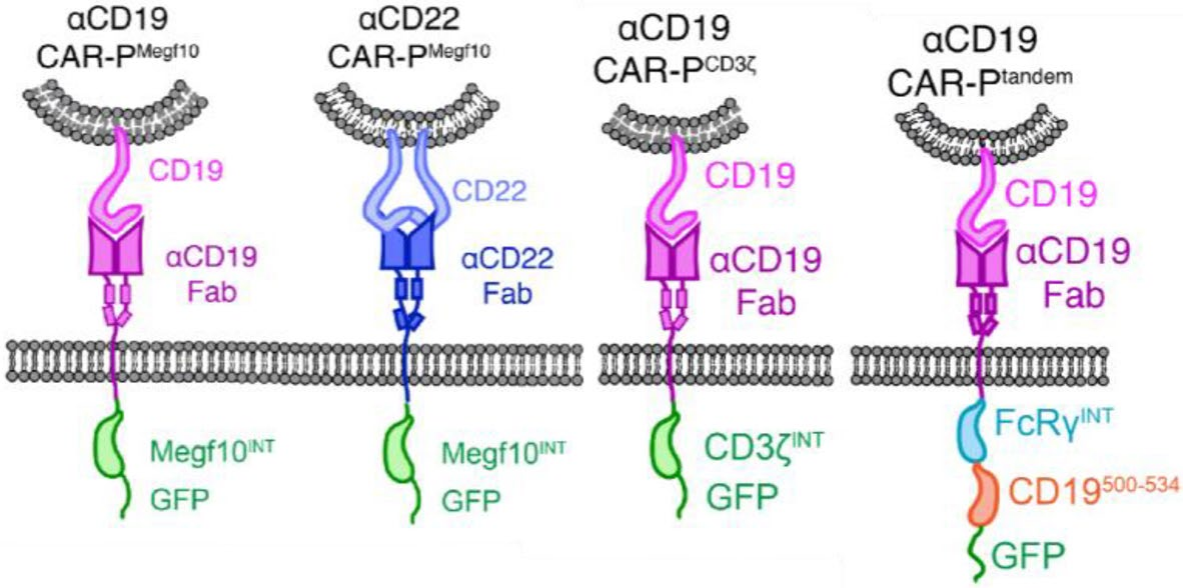
The structure of CAR-P constructs [22]. Copyright©2018, Morrissey et al.

Exploiting the robust phagocytic capability of macrophages to kill tumor cells is very attractive in developing new immunotherapies, but the specific recognition is first to be addressed. The initial impact of antibody-dependent cellular phagocytosis (ADCP) was demonstrated. However, it was not widely accepted as a potential treatment because it potentially triggered the immunosuppressive signaling or activated the pro-tumor macrophage subsets [30]. Engineering macrophages with CARs endows the cells with improved anti-tumor capabilities and new targeting moieties, proving a new promising immunotherapy for solid tumors [31]. CAR typically consists of a solitary scFv, which attaches to antigens outside the cell, a hinge domain, a transmembrane domain (usually CD8), and an intracellular domain (such as CD3ζ) for facilitating costimulatory and active signaling. As a pioneering work, Morrissey and colleagues constructed a family of CAR molecules containing the cytosolic domains from murine phagocytic receptors involving Megf10, FcRɣ, CD3ζ, Bai1, and MerTK, as the intracellular signaling domains [22]. The findings indicated that the engineered CAR-Ms facilitated the direct engulfment of particles or cancer cells that were specific to the antigen, resulting in a reduction of the tumor load by more than 40% (Fig. 1). To conquer the tumor-penetrating problem that precludes CAR-T immunotherapy, Zhang et al. developed a novel murine CAR macrophage harboring mouse CD147 transmembrane and intracellular regions, which enables macrophages to cross the extracellular matrix (ECM) to target HER2^+^ cancer cells [23]. Thus, the original CAR macrophage engineered with prototypic CARs is armed with specific tumor recognition and improved phagocytosis or other macrophage unique functions.

**Fig. 2:**
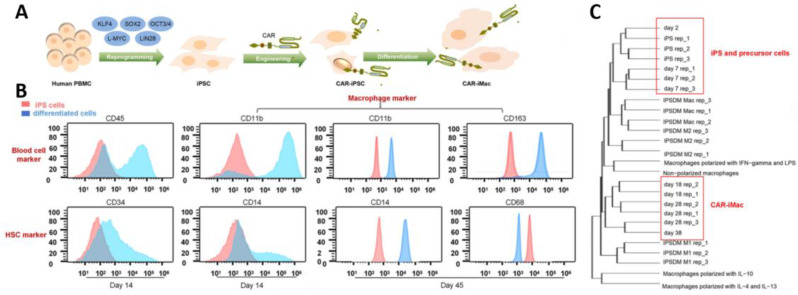
CAR-macrophage cells derived from pluripotent stem cells (CAR-iMacs) with antigen-dependent anticancer properties [24]. (**A**) An outline of the procedure for creating CAR-iMacs using CAR-iPSCs. (**B**) Flow cytometry was used to analyze cells differentiated at various stages from iPSCs. (**C**) A cluster analysis was performed on transcriptomes derived from CAR-iPSCs, differentiated cells, primary macrophages, and untransduced macrophages derived from iPSCs. Copyright©2020, Zhang et al.

Above is CAR-M related investigation in the murine context, later the researchers explored CAR-M therapeutic potential in humans. CAR molecules constructed by Klichinsky et al. include immunoreceptor tyrosine-based activation motifs (ITAMs) within their intracellular domain CD3ζ. They demonstrated that the phagocytic capability of macrophages was significantly enhanced when CD3ζ-based CARs were introduced into human macrophage THP-1 cells or primary human monocyte-derived macrophages [32]. However, to tackle the restricted growth capacity of macrophages, Zhang et al. took a different approach and developed a novel strategy to generate CAR-expressing macrophages (CAR-iMacs) from human pluripotent stem cells (iPSCs) [24] (Fig. 2). The constructed CAR-iMacs demonstrate specific targeting CD19^+^ or mesothelin^+^ cancer cells, enhanced phagocytosis and pro-inflammatory M1 polarization.

Nowadays, the advent of innovative technologies including synthetic biology, genetic and epigenetic editing, and biomaterial platforms promotes the development of more sophisticated designed CAR-engineered macrophages [33]. CAR-M therapy is being developed to remove roadblocks that hinder its efficacy with multiple strategies. For instance, the optimized CAR structure was generated by incorporation of the immune-stimulatory domains such as PI3K recruiting domain CD19^500–534^ and IFN-γ activating domain [22, 34]. Also, nanoparticles were coupled with the inhibitors of checkpoint CD47 to generate a new generation of CAR-Ms, leading to enhanced phagocytosis and locoregional responses to solid cancers [35, 36]. More recently, RNA-editing and metabolic reprogramming platforms were introduced in CAR-M design, empowering the engineered macrophages with more potential to combat cancers [37, 38].

## Practical application of CAR-M treatments in a clinical setting

The mounting evidence demonstrating the effectiveness, security, and practicability of CAR-M therapy is fueling the rising excitement to initiate the clinical trial of this treatment. Currently, there are seven CAR-M cell therapy candidates in various stages of preclinical and clinical research and development (Table 1). Out of these, the FDA has approved two CAR-M clinical trials, while three clinical trials have obtained licenses to assess their effectiveness in treating solid tumors, up until November 2020 [23, 39.

**Table 1:**
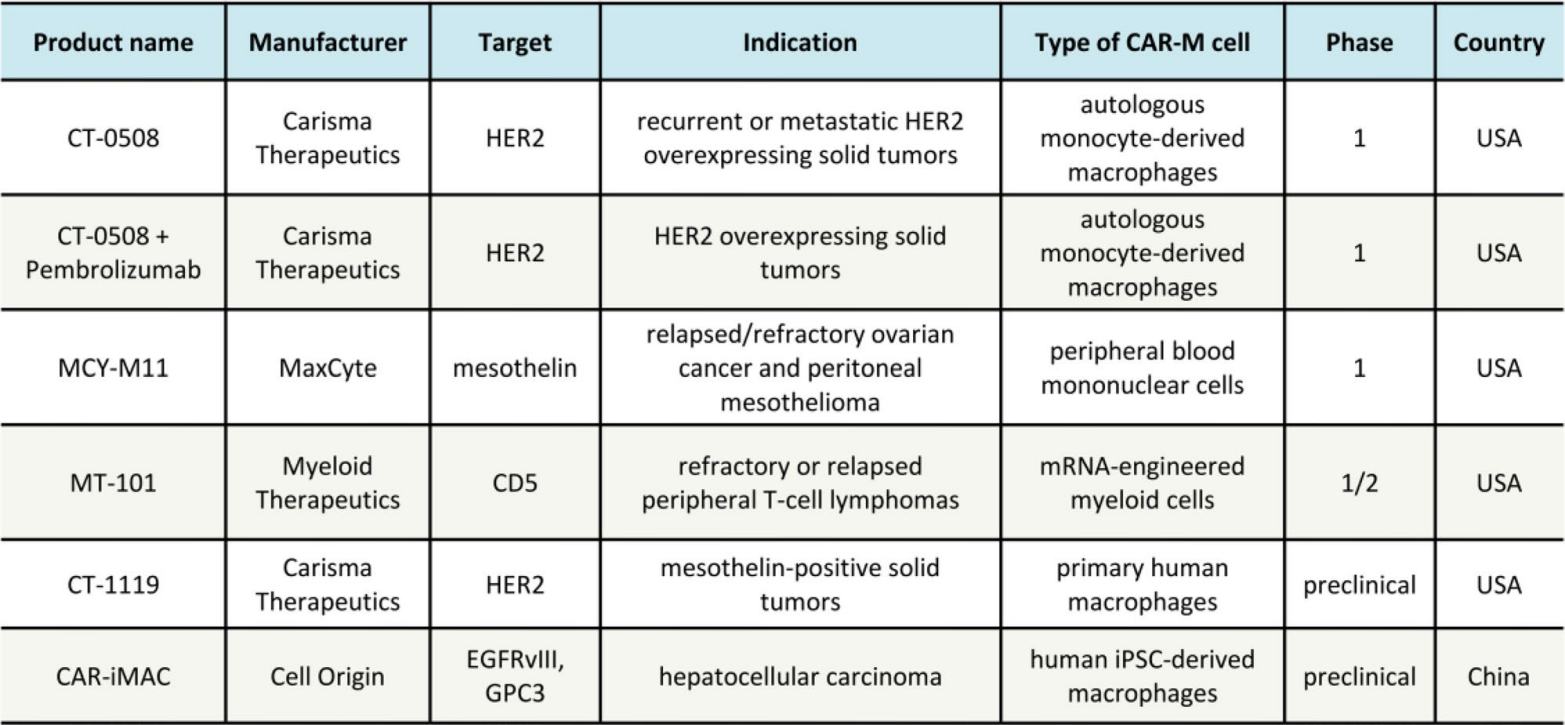
CAR-M-based clinical trials

CT-0508, a CAR-M therapy developed by Carisma Therapeutics, has received approval to commence clinical trials for the treatment of patients with recurrent or metastatic solid tumors that overexpress HER2 (NCT04660929) [40]. The adoptive therapy is focused on patients with no available approved HER2-targeted therapies or with no response to treatment. Preclinical experiments have shown that the engineered CAR-Ms effectively penetrated into tumors, reshaped the tumor microenvironment (TME), and triggered adaptive anti-cancer reactions, suggesting their ability to address the main obstacles encountered by CAR-T cell treatments in solid tumors [41]. Preliminary data reveals that CT-0508 is generally safe and well-tolerated with no dose-related toxicities. In addition to the monotherapy, Carisma has started a clinical trial to examine the possible supplementary impacts of the combined treatment of CT-0508 and the Pembrolizumab checkpoint inhibitor. Furthermore, this company is currently in the pre-clinical pipeline for CAR-M CT-1119, which involves ex vivo gene-modified autologous CAR-Macrophages that specifically target solid tumors expressing mesothelin.

MaxCyte’s drug candidate, MCY-M11, obtained prior approval to commence a Phase l clinical trial targeting patients with relapsed/refractory ovarian cancer and peritoneal mesothelioma (NCT03608618) [42]. MCY-M11 is created by modifying human peripheral blood mononuclear cells (PBMCs) with the mesothelin-targeting CAR mRNA [43]. The production of MCY-M11 exploited MaxCyte’s proprietary Flow Electroporation® technology enabling rapid manufacture and delivery back to the patients bypassing viral components or cell expansion. Promising results from single-round administrations of MCY-M11 are motivating and justify the investigation of supplementary approaches like prior chemotherapy and repeated cycles to enhance effectiveness.

NCT (05138458) is the identifier for MT-101, an mRNA-engineered CAR-M therapy developed by Myeloid Therapeutics. It has obtained fast-track designation from the FDA to treat patients with relapsed or refractory, CD5-positive peripheral T-cell lymphoma (PTCL) [44]. The production of MT-101 involved the utilization of myeloid cells obtained from the patient’s bloodstream and its purpose was to selectively focus on CD5, a surface receptor present in over 75% of PTCL cases. Currently, the safety, tolerability, and efficacy of MT-101 are being assessed in the ongoing Phase 1/2 clinical trial, expected to conclude in October 2024.

Unlike the majority of current CAR-M therapies derived from fully developed immune cells, CAR-iMAC is generated from induced pluripotent stem cells that have been modified with CAR molecules and subsequently transformed into specialized macrophages (iPSCs) [24, 45]. The approach provides an efficient platform for the ex vivo production of macrophages in sufficient quantities. When combined with CD47 antibodies, the most recent preclinical findings revealed that CAR-iMAC therapy resulted in a significant regression of hepatoma cells in mice.

## The principles of CAR-M design

The effectiveness of CAR-M treatments relies on various factors associated with the CAR elements, the administration of CAR molecules, the origin of modified cells, and the in vivo durability and growth, among others [7, 46].

### Enhancing the CAR intracellular signaling

**Fig. 3:**
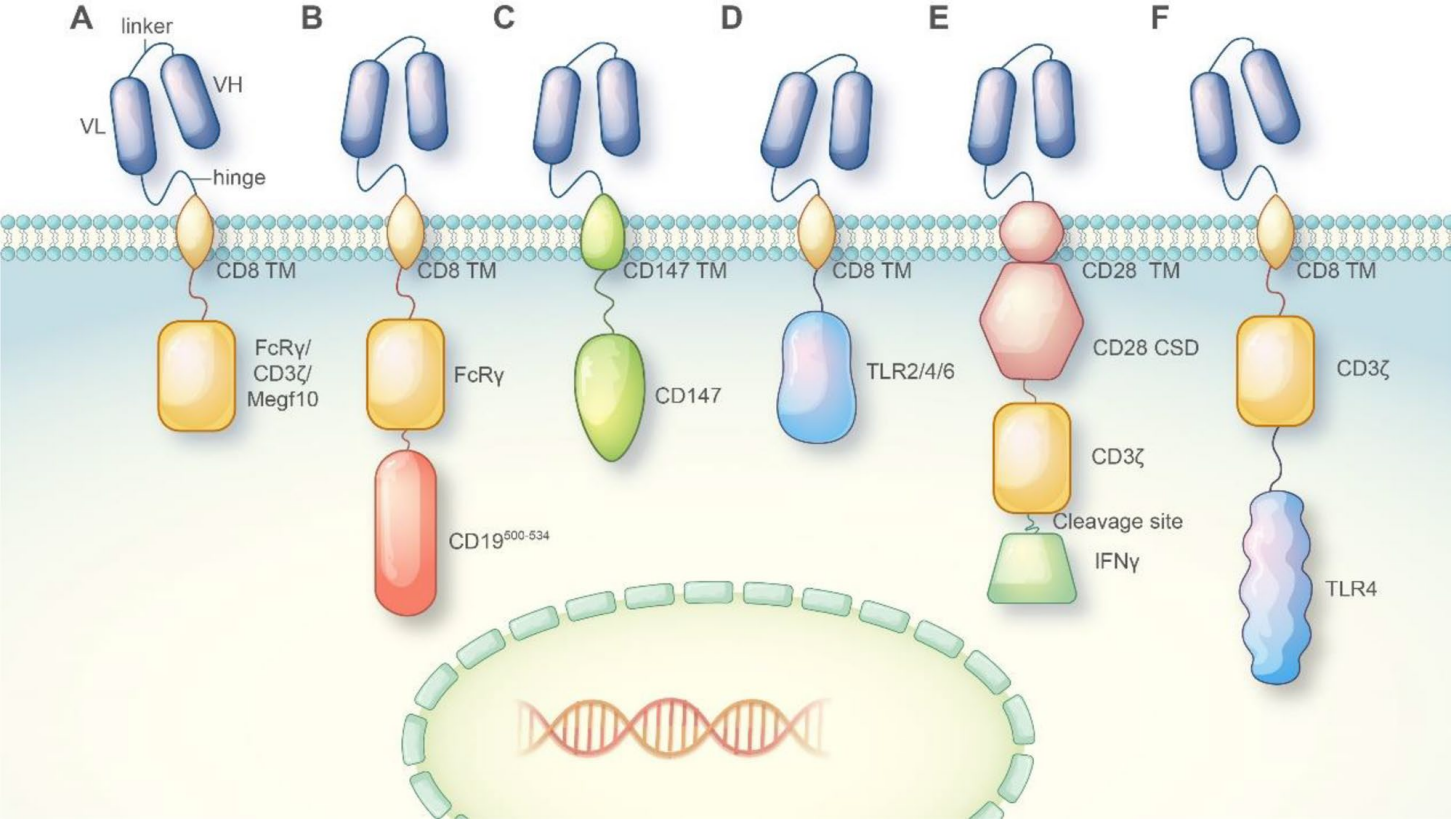
Element composition of different CARs in CAR-macrophage design. The current design of the CAR-macrophage molecule includes the extracellular scFv antibody, the hinge region, the transmembrane region, and the intracellular activation domain. Various intracellular activation domains were utilized to create CAR-M with diverse anti-tumor capabilities. (**A**) CAR-M incorporating phagocytosis domains such as FcRγ /CD3ξ/Megf10 demonstrated the ability to engulf antigen-specific target cells and impede tumor advancement. (**B**) The tandem of FcRγ and PI3K recruiting domain resulted in the engulfment of entire cells. (**C**) The transmembrane region and intracellular domain responsible for activation of CD147 used in CAR-M can secrete matrix metalloproteinases, which help immune cells infiltrate. (**D**) Receptors involved in the transduction of inflammatory pathways were incorporated into CAR-M to induce M1-type polarization, leading to the manifestation of anti-tumor effects. (**E**) The tandem CAR molecule comprising the costimulatory domain CD28, the phagocytosis domain CD3ξ, and the M1-type cytokine IFN-γ released through a cleavage site exhibits the ability to suppress tumor progression through both phagocytosis and pro-inflammatory anti-tumor effects. (**F**) The co-expression of CD3ξ and TLR4 intracellular domain enhanced the ability to engulf target cells and promoted M1 polarization in an antigen-dependent manner.

The basic elements of traditional CAR molecules consist of an extracellular scFv, a hinge and transmembrane region, and intracellular activation and co-stimulatory domains. The scFv portion is situated on the surface of the CAR cell and linked to the VH and VL regions of a tumor antigen-targeting antibody through a connecting sequence. Typically, the hinge and transmembrane sections originate from CD8 or CD28 sequences, while the majority of the intracellular activation domains consist of CD3ξ and 41-BB or CD28 domains [47]. The design of first-generation CAR-M cells learns a lot from that of CAR-T cells, which however needs to be refined and innovated for improved CAR-M therapy (Fig. 3).

To choose the optimized signaling components in CAR, Morrissey and colleagues introduced the cytosolic domains from different phagocytic receptors to compare their effects on macrophage phagocytosis. The results showed that Megf10 and FcRγ domains rendered the CAR cells to effectively engulf antigen-labeled beads, while Bai1 and MerTK failed to induce cellular phagocytic activity. CD3ξ, a prototypic intracellular domain of CAR-T, was revealed to have a comparable potential with FcRγ [22]. Like this finding, Wenyan et al. found that the CAR macrophages harboring the MerTK domain were unable to induce the immune response to invading pathogens [48]. However, there was another report indicating the instructive role of the MerTK domain in macrophages’ response to target cells [25], implying that the intracellular signaling domain may have a distinct role in a given setting. CD3ξ, due to its outstanding signaling transducing ability, has now been utilized in most CAR constructs for macrophage engineering [41].

Furthermore, apart from the inherent elements of CARs, the scientists contemplate incorporating the inflammatory signaling domains into the design of CARs, potentially facilitating the transformation of a ‘cold’ tumor environment into a ‘hot’ one [49]. To address this issue, Townsend and his team developed an innovative CAR macrophage system (MOTO-CAR™) that integrated the intracellular signaling domains of TLR4 or TLR2 into the CAR frame [50, 51]. This modification led to a notable increase in phagocytosis and enhanced effectiveness of CAR-Ms. Consistent with this, another research demonstrated that the CARs incorporating TLR4 and/or IFN-γ receptor domains resulted in enhanced production of CD86, MHC-II, and TNF-α by macrophages, which are characteristic features of M1 phenotype, and accelerated tumor regression [52]. Comparable findings were also observed in macrophages that were transfected with the intracellular portions of CD3ξ and IFN-γ in succession, or CD3ξ alone [34, 45]. A recently developed second iteration of CAR-M, known as CAR-M2, demonstrated enhanced tumor cell elimination and modulation of the tumor environment. CAR-M2 is constructed from induced pluripotent stem cells and features an intracellular domain combining CD3ζ and TLR4 [53].

### De-repressing the phagocytosis checkpoint

The optimal CAR-M design is aimed at maximizing macrophage phagocytosis for tumor eradication. However, the ability of macrophages to phagocytize is significantly hindered by the phagocytosis checkpoint, also known as the signal that instructs them not to consume. Studies have indicated that the reduction of signal regulatory protein alpha (SIRP-α) on macrophages can cancel out the impact of CD47, a widely recognized checkpoint that is mainly expressed by tumor cells [54, 55]. Inspired by this, Sloas et al. exploited the CRISPR/Cas9 approach to deplete SIRP-α from primary human macrophages, which were simultaneously engineered with an anti-HER2 CAR to generate CAR-M therapy. The outcome indicated that CAR-Ms lacking SIRP-α displayed an increased secretion of cytokines/chemokines, polarization towards proinflammatory macrophages, and improved anti-cancer responses [56]. As a result, the pairing of magrolimab, an anti-CD47 antibody, with trastuzumab, an anti-HER2 antibody, greatly accelerated the removal of breast cancer cells that have HER2^+^ receptors [57]. Of interest, a recent study demonstrated that CD47 blockade in combination with radiotherapy induced a systemic and robust macrophage response against tumors. In addition to locally activating macrophages, CD47 blockade also triggers the abscopal effect by activating macrophages that move into tumor sites not exposed to radiation due to inflammation caused by radiation [58].

Similar to intratumoral T cells, recent evidence indicates that TAMs also possess the programmed cell death protein 1 (PD1), which is a crucial immune checkpoint receptor, when engaged with the cognate ligand PD-L1 that is dominantly expressed on tumor cells, would induce an inhibitory signaling for cancer immune escape. Consequently, the inhibition of PD1 and PD-L1 interaction greatly enhanced in vivo macrophage phagocytosis, suppressed tumor development, and prolonged the survival of mice with tumors [55]. In a CT26 mice model, a colorectal carcinoma believed to be unresponsive to anti-PD1 monotherapy, the combination of anti-HER2 CAR-M (CT-0508) treatment and pembrolizumab, a PD1 checkpoint inhibitor, demonstrated enhanced TME activation and improved therapeutic effectiveness [59]. Carisma Therapeutics, feeling motivated by this development, received authorization to initiate the Phase 1 clinical study aimed at evaluating the effectiveness and safety of a combination therapy involving CAR-M and T cell checkpoint inhibitor (Pembrolizumab) for the treatment of recurring or metastatic cancers (NCT04660929).

In addition to these canonical checkpoint molecules, an expanding spectrum of phagocytosis checkpoints are nowadays discovered, some of which exert important roles in regulating macrophage cytotoxicity when incorporated into CAR structure [60, 61]. Recently, the SLAM family receptors were discovered to function as signals that inhibit macrophage phagocytosis by preventing them from engulfing cells. The combination of the antibodies targeting SLAMF2 and/or SLAMF3 with CD19-specific CAR-Ms significantly increased macrophage phagocytosis and tumoricidal activity [62]. Recently, Wu et al. raised a novel immunotherapy strategy that combined cancer desialylation with iPSC-derived CAR-macrophages (CAR-iMacs) therapy. By administration of blockades of Siglec-5 and Siglec-10, the major siglecs expressed on immune cells mediating the inhibitory signaling upon engaged with sialic acid ligands on tumors, the strategy markedly enhanced cancer cell killing of CAR-Ms and prolonged survival in ovarian cancer mouse models [63].

### The delivery of CAR molecules

#### Viral vector-based CAR delivery

The viral vector system, mainly consisting of lentivirus and adenovirus, is widely used to deliver the CAR molecules for engineering immune cells. However, macrophages are generally refractory to virus-mediated gene transfer [64], and the purifying and concentrating process to obtain a higher titer of virus may cause large amounts of protein impurities and hence serious cytotoxicity. To resolve this issue, Bobadilla et al. developed a new lentiviral vector system that was inserted with Vpx, a virion-packaged accessory protein to improve the transduction efficiency of myeloid cells [65]. Recently, Vpx-containing chimeric lentivirus has been constructed for CAR gene infection, which significantly increased infection efficiency [66].

Adenoviral vector is another well-established method for transferring genes of interest to target cells, particularly in human primary cells [67]. Most adenoviruses currently in use are adenovirus serotype 5 (Ad5), which mainly recognizes the coxsackie-adenovirus receptor, a receptor rarely expressed by many cells. Scientists modified the vector system to construct adenoviruses with the chimeric type 5 and 35 fiber proteins (Ad5/F35), overcoming the cell tropism of Ad5 and elevating the gene transfection efficiency. The engineered macrophages utilized the modified Ad5/F35 adenoviral vector to transport the CAR molecule into primary human macrophages, resulting in enhanced CAR expression and heightened anti-tumor activity [22, 68, 69] (Table 2).

**Table 2:**
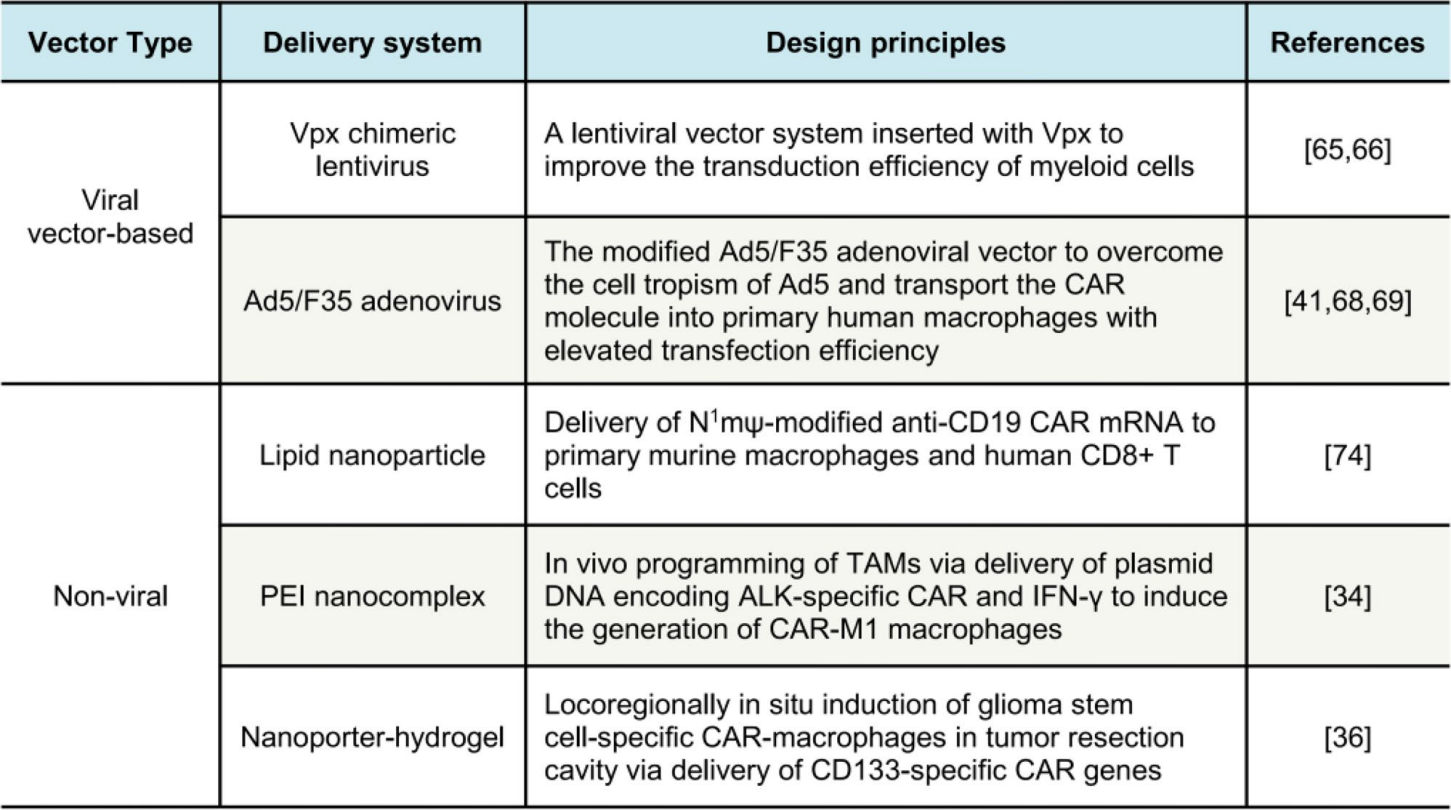
Novel delivery systems of CAR-M cells

#### Non-viral transfer of CAR molecules

Despite the extensive utilization of viral vector-based gene transfer in immune cell gene editing, the ex vivo production of CAR-Ms using viral vectors is both expensive and time-consuming. Moreover, the oncogenicity potential linked to the utilization of a viral carrier restricts its wider clinical implementations [70, 71]. The scientists therefore sought to develop non-viral approaches for transferring the CAR molecules, though it is truly a hard task for macrophages.

**Fig. 4:**
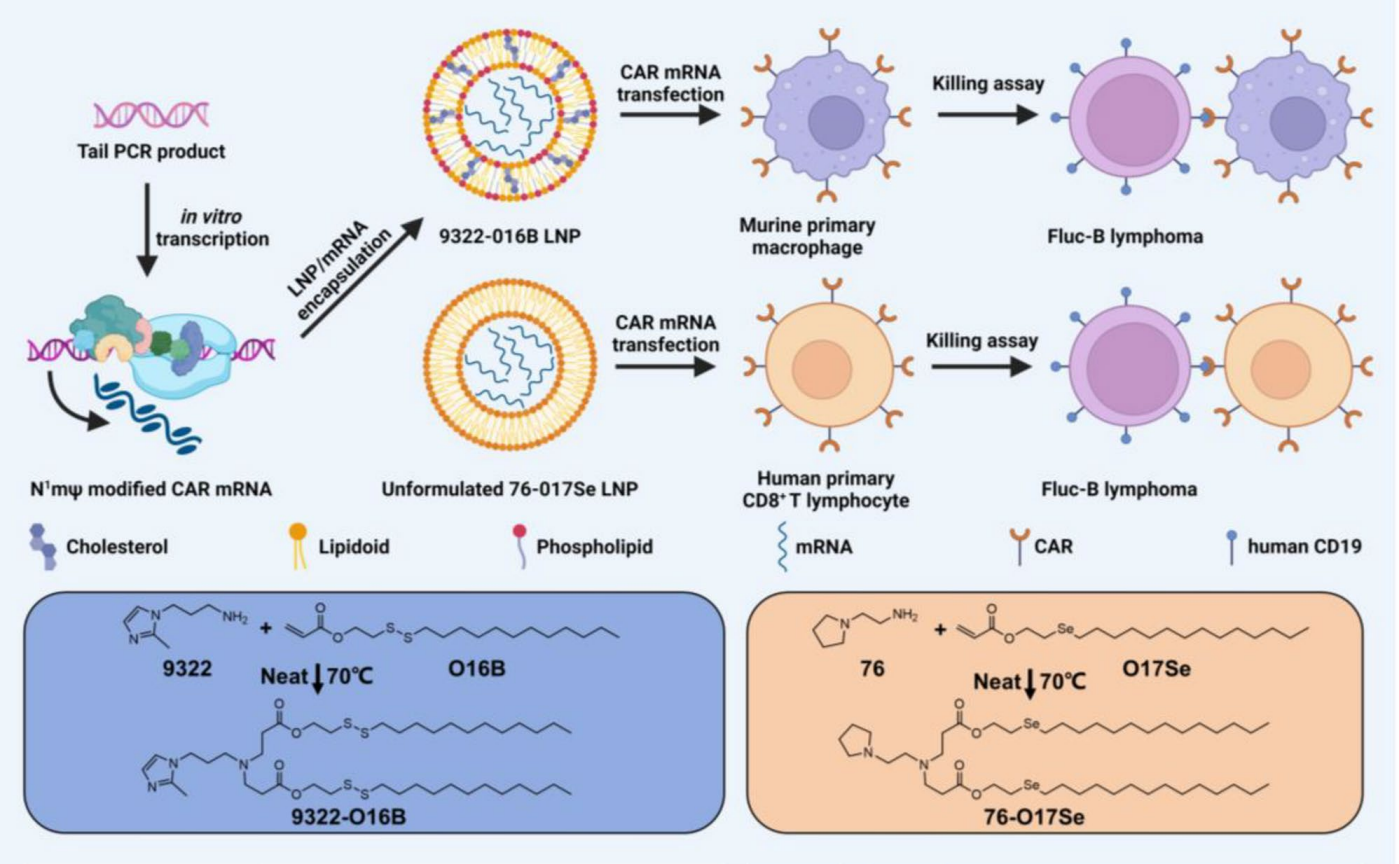
In vitro system using lipid nanoparticles to deliver mRNA to macrophages and T cells with chimeric antigen receptor [74]. Illustration depicting the delivery of N^1^mψ-modified CAR mRNA to primary murine macrophages and human CD8^+^ T cells. Reproduced with permission. Copyright©2022, American Chemical Society.

Non-viral gene delivery is generally thought to introduce genetic materials via cell membranes, and leveraging biocompatible materials to deliver the gene editing agents has emerged as a novel strategy to implement immunotherapy [72, 73]. CAR gene delivery using cationic polymers and lipid nanoparticles (LNPs) has gained considerable attention in recent years. In an attempt, Ye et al. fabricated the LNPs to deliver CD19-targeting CAR mRNA to mouse macrophages and human T cells [74]. They screened a set of mRNA and lipids (Fig. 4) to reveal that the incorporation of phospholipids (DOPE) into LNP was essential for the delivery of the nucleic acid. Both LNP formulations and mRNA modifications were optimized for in vitro mRNA transfection and subsequently cytotoxic effects on B lymphoma cells. The data paves the way to utilize the nanocarriers to deliver CAR molecules into immune cells.

Encouraged by this, a plethora of experiments are underway to deliver CAR molecules with delicately designed biomaterials [73, 75]. In an attempt to deliver macrophage-targeting CAR molecules, polyethylenimine (MPEI) was created to encapsulate the CAR plasmid DNA via electrostatic interaction [34]. Also, a CAR gene-laden nanocomplex was choreographed to send the ErbB2-targeted CARs into intratumoral macrophages [76]. A nanoporter-hydrogel superstructure was designed to deliver glioma stem cell (GSC)-specific CAR gene [36]. These studies support non-viral methods, specifically, nanocarrier-based gene delivery, which are effective for CAR-M editing and fabrication. Considering the additive properties such as low cost, higher efficiency, and diversity of biomaterials, this non-viral approach may offer an opportunity to produce “on-the-shelf” CAR-M therapy (Table 2).

### The manufacturing of CAR macrophages

While much attention to CAR-M therapy is focused on improving the efficacy, where and how to get enough macrophages for immune engineering is another critical issue. CAR-M studies have extensively investigated a range of macrophage sources, such as THP-1 or RAW264.7 cell lines, peripheral blood (PB), bone marrow, umbilical cord blood (CB), and induced pluripotent stem cells (iPSC) (Table 3).

**Table 3:**
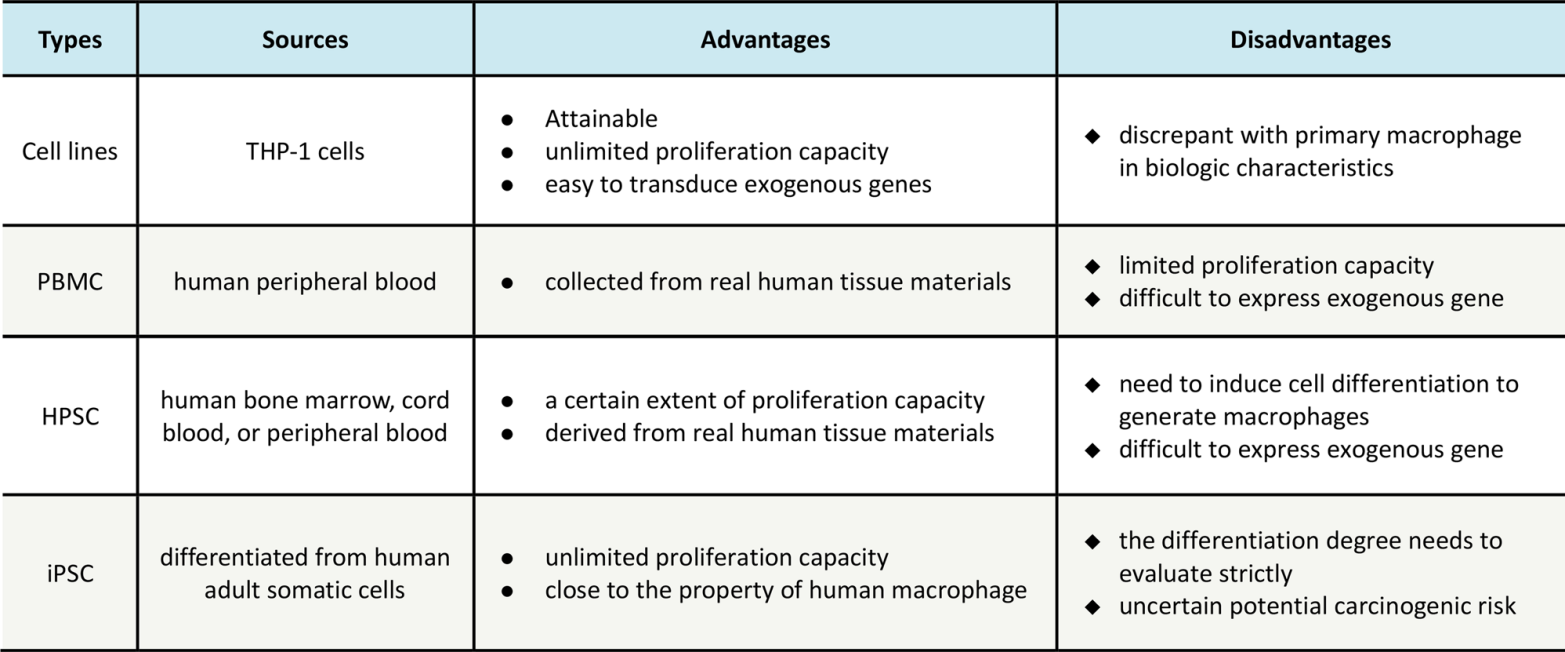
Manufacturing sources of CAR-M cells

#### Cell lines for generating CAR-Ms

CAR macrophages have been created using monocyte/macrophage cell lines due to their capacity to expand and the ease of their cultivation process. Murine macrophage-like cell line RAW264.7 was initially utilized to produce CAR-edited macrophages [23, 25]. In addition, CAR molecules were introduced into BMDMs derived from murine bone marrow to evaluate their phagocytosis and tumoricidal activity in both in vitro and in vivo settings [22, 34].

In humans, leukemia monocytic cell line THP-1 was applied to prepare CAR-Ms in some of the studies to evaluate their anti-tumor potential. The engineered macrophages demonstrated to specifically recognize the antigens such as CD19, HER2, or CEA, engulf tumor cells and impede tumor growth [36, 77]. Although the positive outcomes are encouraging, it is important to acknowledge that the immortalized THP-1 cell lines differ from primary macrophages in terms of their functional traits. For instance, THP-1 cells are generally more sensitive to LPS stimulation with more readiness to induce NF-κB signaling when compared with primary macrophages [78].

#### PBMC-derived macrophages

An advancement in the clinical application of CAR-M therapy involves producing and modifying primary human monocytes/macrophages instead of utilizing cell lines. In autologous cell therapies, primary monocytes are typically cultivated with granulocyte-macrophage colony-stimulating factor (GM-CSF) to induce the transformation of monocytes into a pro-inflammatory phenotype [41]. Alternatively, CAR^+^CD14^+^ monocytes were prepared in a rapid, the same-day protocol, which were differentiated into M1 CAR-M. Since the first PBMC-derived CAR-Ms made by Biglari et al. in 2006, there have been several studies on CAR-edited monocytes/macrophages, which show the potent anti-tumor activity of these engineered cells [41, 79, 80]. Despite the attractive attributes, some obstacles, such as a limited source of healthy blood, relatively lower yield of monocytes, and batch-to-batch variability, are associated with PBMC-derived CAR-macrophage and need to be addressed.

#### HSPCs-derived CAR-Ms

To overcome the scarcity and limited ability of mature macrophages, scientists are striving to produce CAR-Ms using human hematopoietic stem and progenitor cells (HSPCs), which possess a strong capacity for proliferation and a primitive phenotype suitable for lentiviral transduction [81]. Hematopoietic stem and progenitor cells (HSPCs) can be obtained from bone marrow, cord blood (CB), or peripheral blood (PB) following G-CSF mobilization. HSPCs can give rise to immune cells including macrophages that are featured with high proliferation and stronger immune tolerance, which makes them a promising source for allogeneic immunotherapy.

Currently, genetically engineered HSPCs have been utilized to generate CAR-Ms that are featured with durable antigen specificity and phagocytosis [82]. For instance, Paasch and colleagues constructed CAR-Ms from cord blood-derived HSPCs, which were engineered with carcinoembryonic antigen (CEA)-targeting CAR via lentivirus-based gene delivery [77]. Zhang et al. developed a platform to produce HSPC-derived somatic macrophages in a large quantity, and they confirmed the anti-tumor efficacy of CAR-Ms against GD2-expressing neuroblastoma and melanoma [83]. Since CB banks are frequently established in many counties for cryopreservation and storage of cord blood, HSPCs, particularly CB-derived HSPCs, might become a promising and feasible source of CAR-M therapeutics. Future studies are required to address the mass production of HSPCs, and potentially a nonspecific cell-damaging effect associated with CAR editing [84].

#### iPSC-derived macrophages

In addition to HSPCs, human induced pluripotent stem cells (iPSCs), with the “off-the-shelf” potential, have garnered more interest in the development of CAR-M immunotherapy [85, 86]. As early as 2011, Snuji et al. pioneered to generate CAR-Ms from human iPSCs that were pre-engineered with CAR molecules, providing an attractive source of immune cells to be used in the clinic. Recently, an innovative method was established to produce CAR-Ms from iPSCs that were derived through reprogramming of PBMCs. The analysis of function indicates that CAR-Ms derived from iPSCs exhibit comparable characteristics, gene expression patterns, and anti-cancer capabilities to fully developed macrophages, endorsing the possibility of utilizing PBMC-derived iPSCs as an authentic reservoir of CAR-Ms. Compared with other source cells, iPSCs possess a variety of advantages, e.g. readiness to gain from adult somatic cells, unlimited cell source, highly self-renewing capability, and easily standardized preparing protocol. Importantly, iPSCs are generally apt to genetic manipulation and hence CAR engineering, making them a better choice than mature macrophages to generate CAR-based therapy [87, 88].

To maximize the potential of iPSCs, scientists created a novel system called iPS-MLs, which enables the generation of myeloid cell lines from iPSCs. The cells possess the ability to live for a long time and grow by receiving stem-related genes like c-MYC, and additionally, they are equipped with CAR molecules [89]. The preliminary data indicate that the CAR-equipped iPS-MLs can accumulate in tumor tissues and hinder tumor growth and dissemination, especially in combination with IFN-β [90].

The promising results encourage the application of iPSC as the source of CAR-Ms, but several issues, particularly the upscaling production of iPSC-derived macrophages (iPS-Mac), need to be addressed. Recently, Ackermann and colleagues developed an industry-compatible 3D culture system to improve the yields of iPS-Mac [91], and they devised a strategy for continuously producing the iPS-Mac populations. In this case, the so-called myeloid cell forming complexes (MCFC) were differentiated from iPSCs and maintained in suspension culture for long-term production of iPSC-Mac [92]. It is expected that the refined manufacturing of iPSC-Mac would be conductive to promote the practice of CAR-M immunotherapy in clinic [77].

## Emerging strategies for the next generation of CAR-Ms

Although there have been notable advancements in recent years, CAR-M therapy is still in its early stages and requires attention to several crucial concerns. Currently, emerging strategies, marrying advanced macrophage biology with innovative technologies, are being developed for the next generation of CAR-M immunotherapy. The CAR-M protocols are intricately formulated to enhance the effectiveness, adequacy, and safety of modified macrophages, while the innovative gene editing and cell engineering technologies promote the next era of immunotherapy to be more adaptable, personalized, intelligent, and with the potential for clinical application.

### Increasing the robustness and fitness of CAR-Ms

With the progression of the genomic screen and genic editing technologies, researchers nowadays have established a network of systems, such as the single-cell genomics method over CITE-seq, modular pooled KI screening, pooled CRISPR screening, and single-cell transcriptome readout, to search for critical factors that may address some of the challenges (e.g. specificity, controllability, flexibility) CAR therapies face [93–96]. Diligent endeavors enhance the understanding of tumor immunobiology, resulting in enhanced CAR design through the identification of newly found elements crucial for better cellular endurance, performance, and health. In particular, the metabolic and epigenetic regulators, along with surface receptors, transcription factors, and signaling molecules, have emerged as important determinants of myeloid and lymphoid function and hence been considered as the candidate components in designing a new generation of CARs immunotherapy [97–99]. Currently, a panel of metabolism-related molecules such as isocitrate dehydrogenase 2 (IDH2), argininosuccinate synthase (ASS), ornithine transcarbamylase (OTC), and PGC-1 have been identified to strengthen mitochondrial fitness, remove the metabolic barrier, and preclude CAR-T exhaustion [100–102].

Despite relatively little data regarding the metabolic regulation of CAR-M strength, recent studies via pooled CRISPR screening identified aconitate decarboxylase 1 (ACOD1), a mitochondrial enzyme for producing itaconic acid (ITA), as a restrainer of mitochondria function [103]. Targeting ACOD1 in myeloid cells promoted the proinflammatory polarization of TAMs, reversed the immune suppressive status, and significantly enhanced human iPSC-derived CAR-iMACs [38, 104]. This regulation is related to the ITA-mediated regulation of TET DNA dioxygenases, which substantially influenced the epigenetic program and hence gene expression profiles of macrophages [105]. Given its critical significance in cell fate decisions, the metabolic-epigenetic machineryhas nowadays been listed as an essential CAR component to improve the efficacy of immunotherapy [106, 107]. In addition to metabolic modification, it was discovered that the combination of LPS and IFN-γ greatly augmented the phagocytic and tumor-destroying abilities of CAR-Ms, along with the upregulation of costimulatory molecules and proinflammatory cytokines [108]. Further research is necessary to discover unique metabolic and/or epigenetic compounds that play specific roles in different cells and contexts, as identical metabolic occurrences can have varying impacts on the anti-cancer abilities of macrophages and T lymphocytes [109, 110].

### Combined CAR-Ms with other immune cell therapies

The dense extracellular matrix of tumors poses a significant challenge for CAR-T therapeutics, hindering the effective infiltration of T cells into solid tumors. Given the ability of macrophages to penetrate tumors and enlist and stimulate T cells, it is logical to simultaneously administer CAR-Ms and CAR-T cells to optimize the effectiveness against tumors [111–113]. In fact, when HER2-targeted CAR-Ms and polyclonal T cells from the donor were given together, they exhibited a more powerful antitumor reaction compared to each treatment individually in a metastatic SKOV3-xenograft mouse model. The collaboration is probably because of the cross-exhibition of cancer antigens by macrophages and their enhancement of T-cell reactions [41]. The evidence indicated that the enhanced production of co-stimulatory ligands by macrophages significantly enhanced the effectiveness and function of CAR-T cells. In return, the activated CAR-T cells released inflammatory factors to further enhance the cytotoxicity of CAR-Ms. This underlies the synergistic effects of the two engineered immune cells designed to target the same tumor antigens [114]. Alternatively, a recent study proposed that CD4^+^ T cells may remotely induce inflammatory cell death and subsequently shift tumor-associated myeloid cells toward tumoricidal effector phenotypes [115].

Recent research has shown that macrophage-supporting CAR-T cells, which produce a local anti-CD47 inhibitor, can effectively enhance the antibody-dependent cellular phagocytosis (ADCP) or antibody-dependent cellular cytotoxicity (ADCC) of macrophages by unlocking their phagocytosis checkpoints. The additional advantages of the combined therapy were further supported by the preclinical confirmation, clearly demonstrating the potential of the combined immunotherapy to effectively fight resistant and aggressive cancer [116]. To provide further assistance, ongoing clinical trials are assessing the combination of CAR-M (CT-0508) and KEYTRUDA® (pembrolizumab), an anti-PD1 therapy, in the treatment of HER2-overexpressing cancer in humans.

### Biomaterial-mediated CAR-M enhancement

To overcome the challenges current immunotherapy faces, biomaterials, such as hydrogels, nanoparticles, and microparticles, have been applied to specifically modulate immune cells and/or reprogram the TME [33, 117]. Coupling the biomaterials with the immune-supporting factors such as cytokines, signaling molecules, or inhibitors of negative factors proves to efficiently reverse the suppressive milieu and boost macrophage therapy efficacy [118]. In a particular case, macrophages were furnished with circular polymeric bags containing the immunostimulatory cytokine, IFN-γ. The modified macrophages demonstrated to continuing release the cytokine and convert into the antitumor phenotypes to promote cancer eradication [119]. The potent M1-promoting activity of IFN-γ further promotes the development of innovative CAR-M that encompasses the IFN-γ activating domain to improve its efficacy.

A wide range of biomaterials are being applied in cancer immunotherapy due to their high efficiency, easy scale-up, low cost, and customizable properties. A panel of innovative approaches, such as a hydrogel enclosing GM-CSF or FLT3L and CpG, PEG-lipid nanodiscs enclosing STING-activating cyclic dinucleotides (CDNs), and a bridging-lipid nanoparticle (B-LNP) conjugating STING agonist and anti-CD47/PD-L1 were recently developed to increase tumor penetration, disrupt phagocytosis checkpoints and induce long-lasting antitumor immunity [120–122]. More recently, a novel mitochondria-targeting strategy was developed by utilizing porous silicon nanocarriers, which led to the proinflammatory transition of TAMs with the interference of mitochondrial function and increased ROS generation. Thus, the good biosafety and versatile loading capability of biomaterial facilitate the innovated engineered macrophages with improved efficacy and specificity against cancers [123]. Further research may be necessary to enhance the production of such systems following good manufacturing practice (GMP) guidelines, using clinical-grade materials, due to the intricate multicomponent compositions in the latest CAR-M therapy.

### In situ gene editing for CAR-Ms

**Fig. 5:**
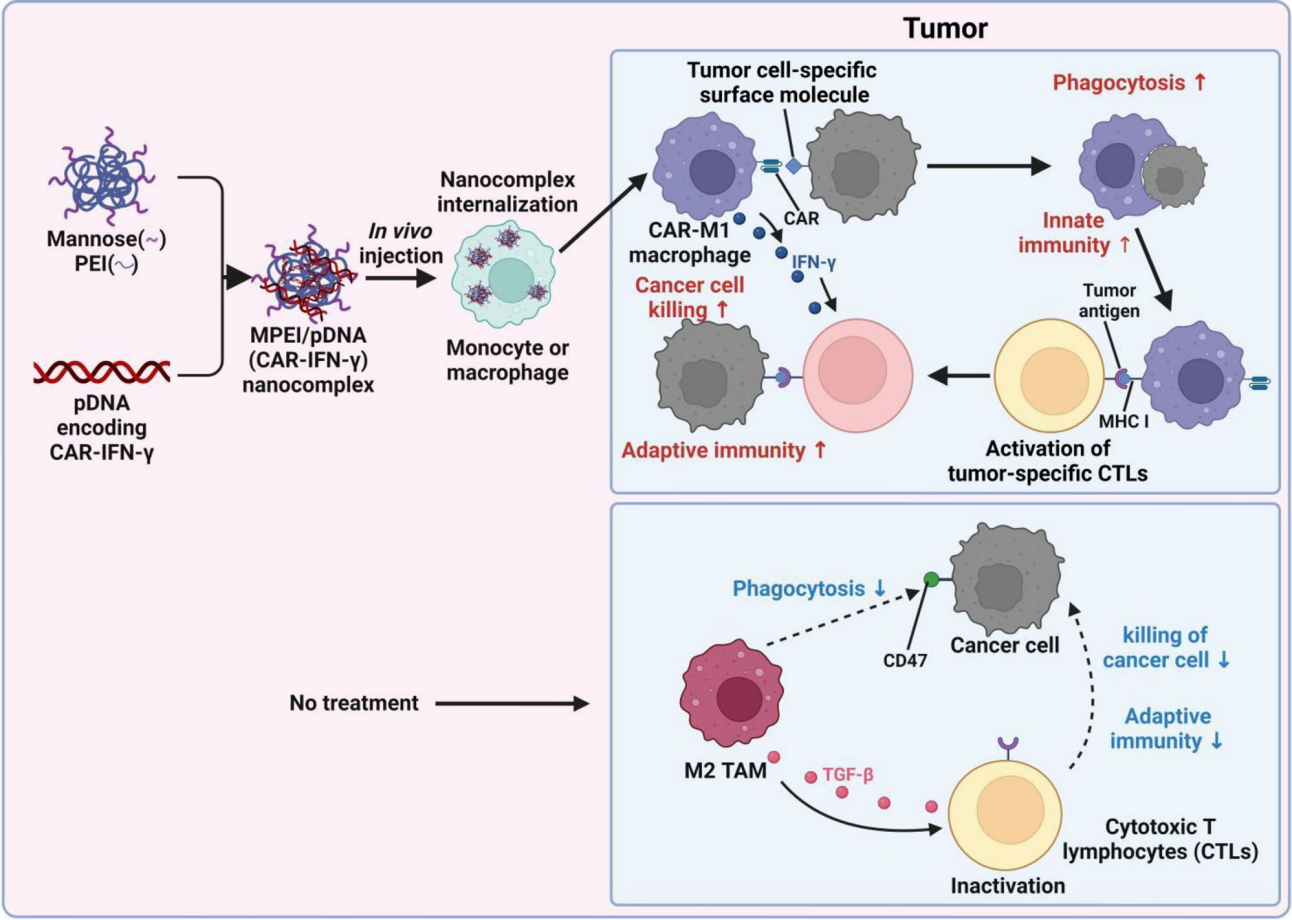
The anti-tumor activity was observed when MPEI/pCAR-IFN-γ nanocomplex was delivered in vivo to create M1 polarized chimeric antigen receptor macrophages [34]. An illustration depicting the delivery of plasmid DNA encoding ALK-specific CAR and IFN-γ (pCAR-IFN-γ) via MPEI to induce the generation of CAR-M1 macrophages in the body. Reproduced with permission. Copyright©2021, Wiley‐VCH GmbH.

In the traditional approach, CAR-Ms are generated by isolating the lymphocytes from the patient, modifying them in a laboratory setting, expanding their numbers, and then transfusing them back into the hosts. Although the protocol is effective in therapy, it is intricate, requires a lot of time and money, and necessitates the use of inventive techniques like in situ immune cell programming to enhance immunotherapy. Emerging evidence suggests that nanomaterials and therapeutic depots have the potential to accumulate in the tumor microenvironment (TME) and selectively target immune cells, thereby providing an opportunity for in vivo manipulation of tumor-associated macrophages (TAMs) and the creation of chimeric antigen receptor-modified macrophages (CAR-Ms) for cancer treatment [36, 124, 125]. A concept of principal evidence, Kang et al. devised a polyethyleneimine (MPEI) decorated with mannose receptors to specifically target macrophages [34], which were then armed with the plasmid DNA encoding anti-ALK CAR and IFN-γ [126–128] (Fig. 5). Administering the MPEI/pCAR-IFN-γ nanocomplex directly into the tumor resulted in macrophages acquiring strong phagocytosis abilities, pro-inflammatory polarization, and modulation of the tumor microenvironment, ultimately leading to significant tumor regression. The study provides a module for the in situ construction of CAR-Ms to elicit anti-tumoral immunity. The nanocomplex preparation is a rapid two-step, charge-induced self-assembly procedure, which could potentially serve as a viable, attainable, and economical pathway to advanced immunotherapy.

**Fig. 6:**
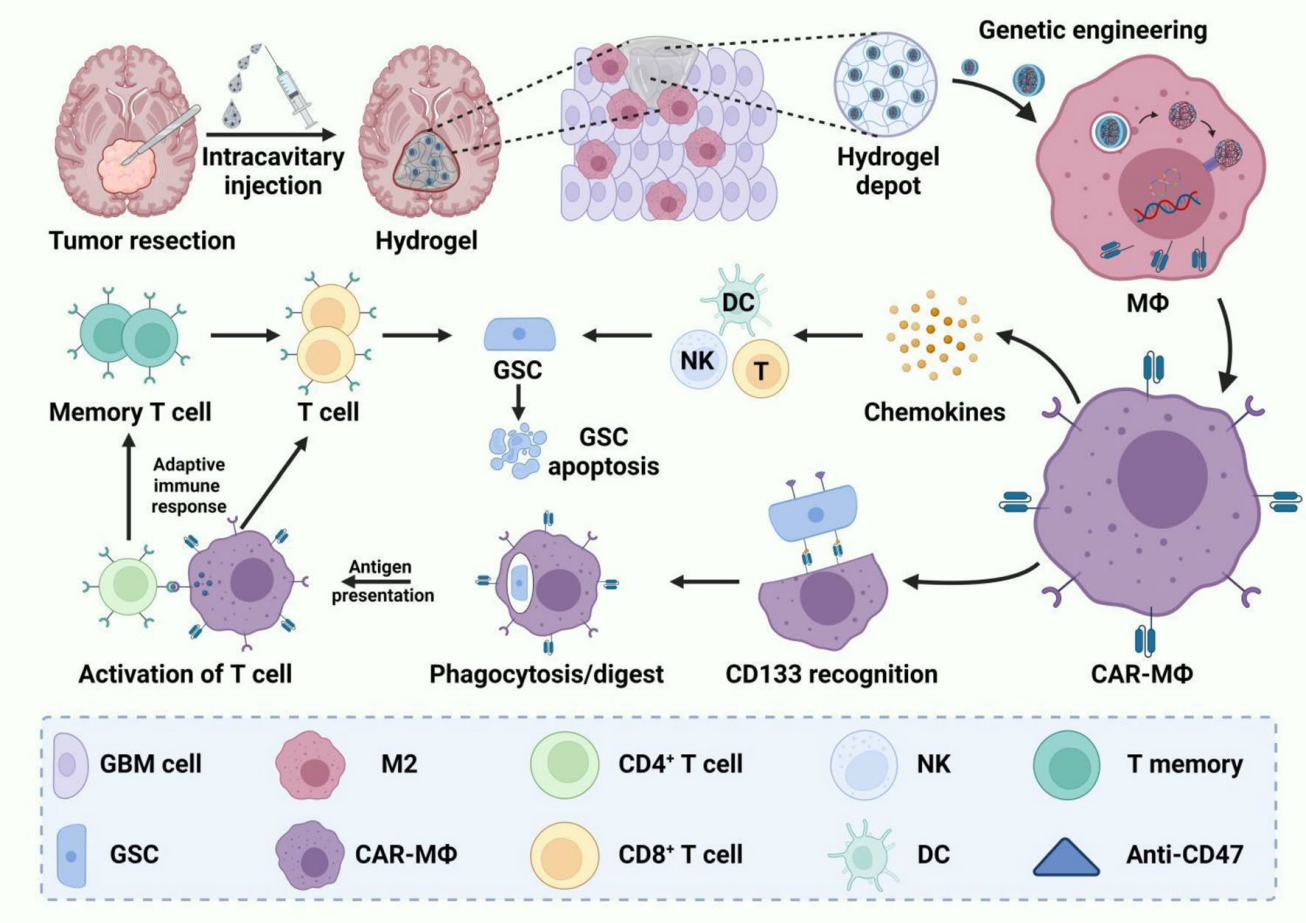
In situ generation of chimeric antigen receptor macrophage with intracavity injected hydrogels primes locoregional anti-tumor immunity [36]. A diagram illustrates the formation of CD133-targeting CAR-Ms around the tumor cavity through the injection of NP-hydrogel superstructures into the cavity, aiming to hinder the reappearance of GBM following surgical intervention. Reproduced with permission. Copyright©2022, The American Association for the Advancement of Science.

In another instance, glioma stem cells (GSC)-targeted CAR macrophages were generated via intracavitary administration of the CAR gene-laden nanoporter, namely NP-CAR complex [36]. For this, DDIKVAV, a brain ECM-derived laminin peptide, and FTKPRF, an immune-stimulating peptide, were conjugated with 9-fluorenylmethyloxycarbonyl (Fmoc) to synthesize peptide-based hydrogels. Negatively charged pCAR was then loaded into the nanomicelle through electrostatic interactions to form the NP-CAR complex (Fig. 6). Local instillation of the CAR-enclosed nanoporter enabled microglial and macrophages to efficiently phagocytize glioblastoma cells and induce long-term antitumor immunity, and the synergic effect was observed when combined with the phagocytosis checkpoint inhibitor, anti-CD47 antibody. Recently, a mucin 1-specific CAR was constructed to engineer the macrophages in tumors. The edited macrophages displayed significantly improved phagocytosis, antigen-presenting activity, and activation of cytotoxic T lymphocytes to combat pancreatic adenocarcinoma (PAAD) [129]. Interestingly, the locoregional CAR-Ms were also generated to induce bactericidal immunologic activity at the disease site [130]. In another strategy, macrophages were equipped with CTLA-4-targeting CARs, combined with a light-responsive biomaterial system containing photoacid and melatonin. Efficiently crossing the blood-brain barrier (BBB), the CAR-M-UZPM system successfully facilitated the in-situ formation of CAR-Ms, leading to the reversal of inflammatory microglia polarization [131].

With its extraordinary efficiency, feasibility, and labor-saving, in situ genetic editing is nowadays used to reprogram macrophage phenotypes and functions beyond direct CAR deliver. Liu et al. developed an innovative approach to reducing the levels of MafB and c-Maf in Kupffer cells (KCs), the predominant group of liver macrophages, by employing a CRISPR/Cas system based on bacteria. The in situ genetic manipulation promoted the inflammatory polarization of KC, induced robust antitumor T cell responses, and hence impeded metastatic liver cancer [132]. To address the issues of poor productivity, lack of specificity, and possible adverse reactions linked to in situ reprogramming of TAM, Zhang et al. devised internally and externally modified exosomes (IEEEs) through CRISPR-based genetic editing of PI3Kγ [133]. Thus, the locoregional in situ method provides a convenient and economical way to genetically reprogram macrophages to transiently express disease-specific receptors without the need to extract and culture cells from patients. With the optimized approach, CAR therapy could be converted from an autologous medicinal product into an off-the-shelf treatment that can be used by anyone.

### mRNA-based CAR gene engineering

The mRNA vaccines for SARS-CoV-2 have sparked a heightened curiosity in the investigation of therapeutics based on mRNA. Over the past few decades, the pharmacological and immunological aspects of mRNA have been extensively optimized for clinical use. In vitro transcribed mRNA approaches have displayed a high safety profile and straightforward producing procedure, but their application is hindered by low stability and transfection efficiency. Coupling lipid nanoparticles (LNPs) with mRNA technology enables the delivery of the molecules encoding therapeutically relevant proteins including CAR molecules and associated immune regulators [134–136].

Margaret et al. conducted groundbreaking research by screening a collection of 24 ionizable lipids, successfully establishing a highly effective method to transport mRNA into human immune cells. The selected lipid nanoparticles (LNPs) enabled T cells to highly express CAR molecules, and efficiently kill cancer cells while maintaining reduced cytotoxicity in normal cells [137]. Similarly, Yang and colleagues developed a distinct lipid nanoparticle (LNP) containing two messenger RNAs (mRNAs) that carried genetic instructions for GPC3-targeting chimeric antigen receptor (CAR) and Siglec-G lacking ITIMs. This innovative design aimed to alleviate immune suppression caused by CD24. The administration of LNP mRNA yields CAR-edited macrophages with robust phagocytic function and tumor-eradicating effects [138]. The emerging data have shown the promise of mRNA-based gene editing in efficiently engineering immune cells, and the development of cell Type-Specific mRNA Delivery is undergoing [139]. Clinical trials now incorporate IVT mRNA technology due to its stability within cells, efficient translation, and minimal immunogenicity. The initial mRNA-modified CAR monocyte MT-101 is presently being tested in a Phase I/II clinical study for refractory or relapsed peripheral T cell lymphoma.

As a promising method for gene editing in living organisms, in vitro-transcribed (IVT) mRNA has been utilized for delivering immune enhancement agents such as co-delivery of immune-stimulatory cytokines, CAR vaccine component, and checkpoint inhibitors [140–142]. For instance, the lipid nanoparticles enclosing CD40 ligand mRNA, a canonical co-stimulatory molecule, in combination with TLR agonists, were developed to reinvigorate the activity of macrophages and other antigen-presenting cells (APCs) [143, 144]. Nanoparticles formulated with mRNAs encoded by interferon regulatory factor 5 (IRF5) and IKK activated by this factor reprogrammed pro-tumor TAMs into anti-tumor types for tumor elimination by Zhang et al. [145]. A recent study has developed an innovative mRNA LNPs that contained the N-terminus of gasdermin (GSDM) to initiate pyroptosis. This resulted in the conversion of immunologically cold tumors into hot ones and the stimulation of anti-tumor immunity [146].

## Challenges and perspectives for CAR-M therapy

### The challenges facing CAR-M therapy

Although CAR-M therapy has shown encouraging outcomes in recent times, the creation of modified macrophages continues to be a difficult task. The major hurdles involve insufficient tumoricidal efficacy, limited expansion capacity, high pliability, and potentially off-target cytotoxicity. Currently, CAR-engineered macrophages display remarkable phagocytic activity and in vitro tumor-killing capability, but they generally exert a mild in vivo effect on tumor progression, not as efficiently as CAR-T cells. Intravenous injection of CAR-Ms, at a limited dose of cells, usually causes limited numbers of macrophages infiltrated in tumors due to their retention in the organs of the lung, liver, and kidney [34, 41, 67]. It was found that peritoneal injection of CAR-M cells largely decreased retention and mostly enriched in tumor tissue [147]. Although local delivery of CAR-Ms exhibits a better effect on tumor suppression compared [148, 149], its inhibition on metastatic tumors is unsatisfactory. In addition, the possibility that CAR-Ms might be remolded into the pro-tumor M2 type by the immunosuppressive microenvironment of solid tumors needs to be carefully evaluated. It is highly recommended to conduct correlative analysis on the immune phenotypes of cancer patients both before and following CAR-M treatment [150]. Furthermore, it has been documented that macrophages play a significant part in the development of cytokine release syndrome (CRS) induced by CAR-T therapy primarily by releasing proinflammatory cytokines like IL-6[151, 152]. To enhance the effectiveness against tumors and minimize cytotoxicity, it is necessary to employ advanced strategies for the integration of CAR-M and CAR-T therapy, as well as other immunotherapies. Besides, heterogeneous antigen levels and even loss of surface antigens on tumor cells may cause the immune escape, setting another formidable barrier for CAR-Ms to precisely and effectively kill target cells as CAR-T cells [153].

### Perspective on the next-generation of CAR-Ms

The clinical setting will witness the development of more intricately designed, built, and evaluated next-generation CAR-M therapeutics due to progress in tumor immunology and advancements in CAR technology. Particular attention should be attached to the key points for improved CAR-M therapeutics, such as improving CAR design to maximize macrophage phagocytosis, combining other treatments to achieve synergistic effects, simplifying and optimizing CAR-M manufacturing process, and generating allogeneic or “off-the-shelf” therapeutic products, etc. [20, 154].

Ongoing research has dedicated significant resources to improving the fundamental composition of CAR molecules, including the hinge and transmembrane regions, to bolster the mechanical strength of CAR molecules and augment the anti-tumor capabilities of CAR-Ms. An examination centered on the interaction between CAR and antigen has the potential to facilitate the identification of CAR cells that possess unique sensitivities towards tumor recognition and function, ultimately paving the way for controlled immunotherapy for cancer through drug intervention [155]. New generations of CAR molecules are currently developed to harbor multiple tandem functional units that encompass specific cytokines, transcription factors, activating signaling molecules, inhibitors of phagocytosis checkpoint, etc. With the new discovery of the epigenetic and metabolic regulators key for macrophage robustness and fitness [38, 156, 157], CAR therapeutic will be armed with more layers of regulatory machinery that are inaccessible for traditional therapeutics. By integrating a comprehensive regulatory network, scientists endeavor to form the AND, OR, and NOT logic gates in new CAR designs to develop a smarter and precisely controlled living therapeutic material [158, 159].

Evidence has shown that iPSC-derived macrophages enable the scalable production of therapeutic cells, although further efforts are needed to improve and standardize the manufacturing protocol for the production of clinical-grade products [86]. Additionally, as pluripotent cells are generally amenable to genetic manipulations, an expanding spectrum of molecules central to CAR-M efficacy are edited and optimized to develop new therapeutics with customized sense-and-respond modules [160]. In this regard, the application of innovative technology such as synthetic biology, in situ gene editing, and mRNA-based cell engineering would provide a potent, effective, and flexible platform technology [46, 161, 162]. At the same time, biomaterials have demonstrated the capability to improve the effectiveness of engineered cells by transporting therapeutic carriers (referred to as ‘backpacks’), or facilitating in situ CAR editing through the delivery of mRNA, DNA, and CRISPR-based gene-editing systems [163]. Considering the uniqueness of biomaterials in translating the disease inputs such as pH, light, and hypoxia into functional outputs like cleavage, gel-sol transition, and conformational change, it is expected that marrying biomaterials with CAR technology will foster smarter, controllable, and cost-effective immunotherapy [164].

Given the combined effect of the innate and adaptive immune response to cancer, it is logical to develop CAR-Ms alongside CAR-T cells, particularly those with increased immunogenicity through tumor vaccines. Recent data have shown that vaccine-boosted CAR-T cells exhibited the robust ability to exuberantly release IFN-γ, deliver the costimulatory signals to and activate innate immune cells, setting the basis for improved immunotherapy [165]. Furthermore, it is worth exploring the potential of investigating the combination of CAR-M with conventional therapies like chemotherapy, radiation, and other targeted drugs. These treatments have the potential to stimulate the release of tumor antigens and damage-associated molecular patterns (DAMPs) when eliminating cancer cells, leading to immunogenic cell death (ICD) and effectively reactivating intratumoral macrophages [166, 167]. Thus, a well-organized CAR-M regimen may include orchestrating multiple therapeutics with optimized doses, schedules, and agents to achieve maximized antitumoral potential and patients’ well-being [168].

The cost and complexity of manufacturing are still the major obstacles of current CAR-based immunotherapy, and the difficulty in the control of dosing and activity of CAR cells, as traditional medicines, impedes their clinical application. To conquer it, a new endeavor is undergoing to develop a drug-like therapeutic that can safely and effectively control CAR cell activity. By establishing high-performance CAR screen platforms, a panel of FDA-approved small molecules was recently identified to serve as the ON/OFF switch for regulating the CAR activity [155, 169, 170]. In the ensuing years, drug-gated CAR circuits would be increasingly emerging to identify specific molecules to precisely control CAR-M activity [171]. It is anticipated that the successful development of injectable drugs may supplant current CAR-M therapy if shown to be equivalently, and “on-the-shelf” therapeutics might be a reality to benefit cancer patients [172].

## Conclusions

Macrophages possess a variety of anti-tumor potentials and the potential for being edited. So far, CAR-M cells constructed using gene editing technology have been shown to exert anti-tumor effects through phagocytosis and inflammatory regulation. Although the FDA has approved two clinical trials of CAR-M, the anti-tumor efficacy of CAR-M still needs to be fully evaluated. The efficacy of CAR-M can be enhanced by designing novel CAR constructs or by combining them with macrophage phagocytic checkpoint inhibitors. In addition, the resistance of myeloid cells, such as macrophages, to exogenous genes can be alleviated by improving the gene delivery system of viral vectors, using non-viral vector delivery methods such as nanoliposomes, and utilizing in vivo mRNA editing technology. HPSCs, especially iPSC-derived macrophages, are becoming the future trend for CAR-M chassis cells. Enhancing the inflammatory signaling and phagocytosis of CAR-M cells by artificially intervening with the metabolic enzymes of macrophages is also an optimization strategy that can be explored in the future. However, the source of CAR-M cells and their anti-tumor properties are still important considerations. By integrating advanced knowledge of TAMs and more sophistically designed CAR engineering strategies, we would expect the advent of a new generation of CAR-M therapy with improved safety, potency, and accessibility for patients to substantially impact human health.

## Data Availability

No datasets were generated or analysed during the current study.

## Abbreviations

ACOD1: Aconitate decarboxylase 1
ADCC: Antibody-dependent cellular cytotoxicity
ADCP: Antibody-dependent cellular phagocytosis
ALL: Lymphoblastic leukemia
APCs: Antigen-presenting cells
ASS: Argininosuccinate synthase
CAR-M: Chimeric antigen receptor macrophage
CAR-T: Chimeric antigen receptor T cell
CB: Cord blood
CRS: Cytokines release syndrome
DAMPs: Damage-associated molecular patterns
ECM: Extracellular matrix
FDA: Food and drug administration
GM-CSF: Granulocyte-macrophage colony-stimulating factor
GMP: Good manufacturing practice
GSC: Glioma stem cells
HSPCs: Hematopoietic stem and progenitor cells
ICD: Immunogenic cell death
IDH2: Isocitrate dehydrogenase 2
IEEEs: Internally and externally modified exosomes
IRF5: Interferon regulatory factor 5
iPSCs: Induced pluripotent stem cells
ITA: Itaconic acid
ITAM: Immunoreceptor tyrosine-based activation motifs
LNPs: Lipid nanoparticles
MCP: Monocyte chemoattractant protein
MMPs: Matrix metalloproteinases
MR: Mannose receptor
OTC: Ornithine transcarbamylase
PB: Peripheral blood
PCM: Plasma cell myeloma
PD1: Programmed cell death protein 1
PTCL: Peripheral T-cell lymphoma
scFv: Single-chain fragment variable
SIRP-α: Signal regulatory protein alpha
SR: Scavenger receptor
TAMs: Tumor associated macrophages
TCR: T cell receptor
TGF: Transforming growth factor
TLR: Toll-like receptor
TME: Tumor microenvironment
TLR: Toll-like receptor
TME: Tumor microenvironment
VEGF: Vascular endothelial growth factors
